# Use of a novel silk mesh for ventral midline hernioplasty in a mare

**DOI:** 10.1186/s12917-015-0379-8

**Published:** 2015-03-13

**Authors:** Jennifer Haupt, José M García-López, Kate Chope

**Affiliations:** Department of Clinical Sciences, Cummings School of Veterinary Medicine, Tufts University, 200 Westboro Road, North Grafton, MA 01536 USA

**Keywords:** Abdominal, Hernia, Hernioplasty, Horse, Mesh, Silk

## Abstract

**Background:**

Ventral midline hernia formation following abdominal surgery in horses is an uncommon complication; however, it can have serious consequences leading to increased morbidity and mortality. Currently, mesh hernioplasty is the treatment of choice for large ventral midline hernias in horses to allow potential return to normal function. Complications following mesh hernioplasty using polypropylene or polyester mesh in horses can be serious and similar to complications seen in human patients, including persistent incisional drainage, mesh infection, hernia recurrence, intra-abdominal adhesions, mesh or body wall failure, recurrent abdominal pain (colic), and peritonitis. This report describes the use of a novel bioresorbable silk mesh for repair of a large ventral midline incisional hernia in a mature, 600-kg horse. To our knowledge, this is the first report of its kind in the literature.

**Case presentation:**

A 9-year-old, 600-kg Warmblood mare presented with a ventral midline hernia following emergency exploratory celiotomy 20 months prior. The mare was anesthetized and a hernioplasty was performed using a novel bioresorbable silk mesh (SERI^®^ Surgical Scaffold; Allergan Medical, Boston, MA). No complications were encountered either intra- or postoperatively. The mare was discharged from the hospital at 3 days postoperatively in an abdominal support bandage. At 8 and 20 weeks postoperatively, ultrasonographic assessment showed evidence of tissue ingrowth within and around the mesh. The mare was able to be bred 2 years in a row, carrying both foals to full gestation with no complications. Following both foalings, the abdomen has maintained a normal contour with no evidence of hernia recurrence.

**Conclusions:**

Ventral abdominal hernias can be repaired in horses using a bioresorbable silk mesh, which provides adequate biomechanical strength while allowing for fibrous tissue ingrowth. The use of a bioresorbable silk mesh for the repair of ventral hernias can be considered as a realistic option as it potentially provides significant benefits over traditional non-resorbable mesh.

## Background

Ventral midline celiotomy is a common procedure for evaluation and treatment of abdominal pain in horses. Early incisional complications following colic surgery include incisional drainage in 29% of horses and subsequent incisional infection in 4% of horses [1]. Long-term complications following celiotomy for surgical treatment of colic in horses include ventral midline hernia formation in 8% to 16% of cases [2,3]. Factors that increase the likelihood for hernia formation include incisional drainage or infection and repeat laparotomy [2-4].

Although not all ventral midline hernias in horses require surgical repair, mesh hernioplasty is the treatment of choice for large defects, which can inhibit athletic activity, gestation and parturition, and can lead to bowel incarceration. Currently, the available mesh implants for use in equine hernioplasty are non-absorbable knitted polypropylene or polyester mesh. Open repair of large ventral midline hernias with subperitoneal mesh placement and hernia ring apposition is the most common surgical technique [5,6]. Major complications can occur in mesh hernioplasty in horses ranging from persistent incisional drainage, mesh infection, hernia recurrence, intra-abdominal adhesions, mesh or body wall failure, recurrent abdominal pain (colic), and peritonitis [5]. Based on the current literature, complication rates following mesh hernioplasty using synthetic, non-absorbable mesh range from 20% to 60%, with mortality rates up to 50% following repair in large horses (>450 kg) [5,7]. The most significant complications leading to increased morbidity and mortality following mesh hernioplasty in horses include persistent mesh infection, rupture of the internal abdominal oblique muscle, and persistent colic [5]. Potential causes of recurrent colic and rupture of the internal abdominal oblique muscle include the rigidity of the synthetic mesh implant and tearing at the mesh-host tissue interface.

Bioresorbable surgical meshes are the ideal implant for mesh hernioplasty as they enhance the mechanical integrity of the body wall while supporting ingrowth of host tissue. In addition, the bioresorbable mesh should reduce postoperative complications including infection and recurrent pain associated with the rigidity of synthetic mesh implants. Recently, a novel bioresorbable silk mesh (SERI^®^ Surgical Scaffold; Allergan Medical, Boston, MA) was evaluated in a rodent abdominal body wall defect model. In this animal model, the silk mesh demonstrated significantly greater ingrowth of fibrous tissue compared with polyester mesh [8]. The silk mesh also provided an ideal bioresorption rate, which allowed the ultimate load to be shared between the body wall, mesh implant, and repair tissue [8]. Based on the promising results of the rodent abdominal body wall defect study, we aimed to evaluate silk mesh for use in a large ventral body wall defect in a large horse with the hypothesis that silk mesh would minimize postoperative complications and provide adequate biomechanical properties during healing. This report describes the use of a novel silk mesh for ventral midline hernioplasty in a large horse.

## Case presentation

A 9-year-old, 600-kg Warmblood mare was admitted for evaluation of a ventral midline hernia. The mare initially presented to the hospital 20 months previously for treatment of abdominal pain (colic) which was diagnosed as a left dorsal displacement of the large colon and was corrected by exploratory celiotomy and reduction of the displacement. Following the exploratory celiotomy, the mare developed an incisional infection and subsequent ventral midline hernia. A previous repair was attempted in the field by a referring veterinarian with no success. In addition, the owner reported no success with abdominal support bandaging.

**Figure 1:**
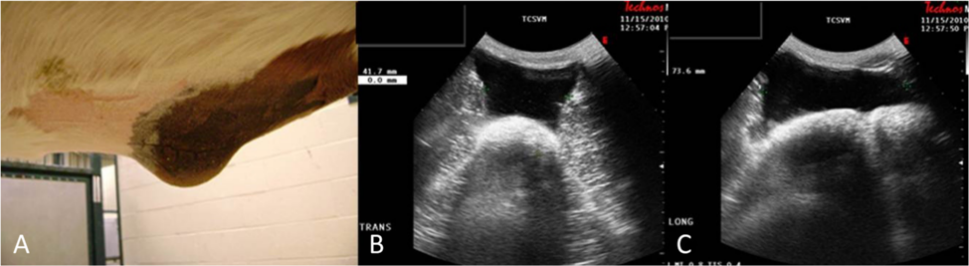
**Preoperative gross (A) and ultrasonographic appearance of the ventral midline hernia using a 5 MHz convex abdominal ultrasound probe in transverse (B) and longitudinal (C) planes revealing peritoneal fluid filling the hernia sac.** Measurement markings on right side of the ultrasound images indicate 0.5-cm intervals. E indicates the cranial and lateral margins of the defect.

On initial evaluation, a ventral midline hernia was palpable at the cranial aspect of the previous celiotomy incision. The hernia sac was soft with a palpable hernia ring and there was no obvious bowel present within the hernia sac (Figure 1). Ultrasonography was used to further evaluate the size of the defect as well as the quality of the hernia ring and surrounding tissues in preparation for mesh hernioplasty. It revealed that the hernia sac contained primarily peritoneal fluid, with the hernia ring being well-demarcated with thick, fibrous edges; the hernia measured 4.2 cm (width) by 7.4 cm (length) (Figure 1). No obvious intra-abdominal adhesions were present either associated with the hernia or surrounding ventral midline. Due to the large size of the hernia defect, the desire to retain the mare for breeding, and the concern about potential herniation of bowel within the hernia sac during gestation or foaling, mesh hernioplasty was elected by the owner.

Surgery was planned for the following day, and food but not water was withheld for 6 hours before anesthesia. Ceftiofur sodium (2.2 mg/kg, IV q12h), gentamicin (6.6 mg/kg, IV q24h), and flunixin meglumine (1.1 mg/kg IV q12h) were administered before surgery and continued for 3 days postoperatively.

### Surgical procedure

The mare was sedated with xylazine hydrochloride (1 mg/kg, IV) and then induced with a combination of ketamine (2.2 mg/kg, IV) and midazolam (0.1 mg/kg, IV). Anesthesia was maintained with isoflurane delivered in 100% oxygen in a semi-closed system. The mare was placed under general anesthesia in dorsal recumbency with routine surgical preparation and draping of the ventral abdomen.

**Figure 2:**
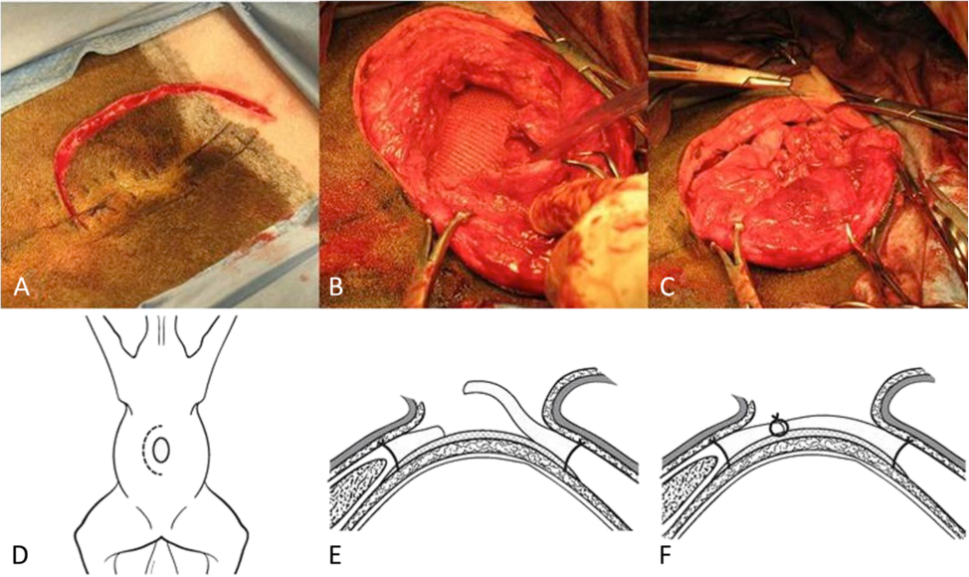
**Intraoperative photographs and corresponding cross-sectional drawings revealing the various stages of intraoperative repair using silk mesh.** Initial elliptical skin incision around the hernia revealing the ventral midline incision from the previous exploratory celiotomy and incision from the previous repair attempt **(A)**. Implantation and suturing of the silk mesh extraperitoneally under the hernia sac **(B)**. Closure of the hernia sac overlying the silk mesh **(C)**. Corresponding figures depict tissue layers **(D–F)**.

A 7-cm semielliptical incision through skin and subcutaneous tissues was made slightly to the right of midline at the edge of the hernia (Figure 2). The fibrous tissue of the hernia was visualized directly deep to the subcutaneous tissue with an approximate thickness of 2 cm. The fascial planes between skin and hernia fibrous tissue were bluntly dissected. A 6-cm semielliptical incision paralleling the skin incision was made through the fibrous tissue of the hernia sac. The peritoneum was visible deep to the hernia but did not appear to be involved or adhered. The body wall was located at edges of fibrous hernia tissue. A 6-cm (width) by 9-cm (length) square of silk mesh (SERI^®^ Surgical Scaffold) was placed in a retroperitoneal position within the hernia ring with pre-placement of 11 individual sutures of #2 nylon encircling the hernia followed by sequential tightening (Figure 2). The fibrous tissue of the hernia sac was closed with 2 polydioxanone (PDS II; Ethicon, Bridgewater, NJ) in a horizontal mattress pattern, interspersed with simple interrupted followed by simple interrupted tacking sutures using 0 poliglecaprone 25 (Monocryl Plus; Ethicon, Bridgewater, NJ), which were placed through the hernia sac and subcutaneous tissue to reduce dead space (Figure 2). Subcutaneous tissue was closed in two layers with 0 poliglecaprone 25 using a simple continuous pattern. The skin was apposed using surgical staples, and the incision was protected in a commercial hernia belt (CM Equine Products, Norco, CA) for recovery. The mare recovered from anesthesia without complications.

### Postoperative care

**Figure 3:**
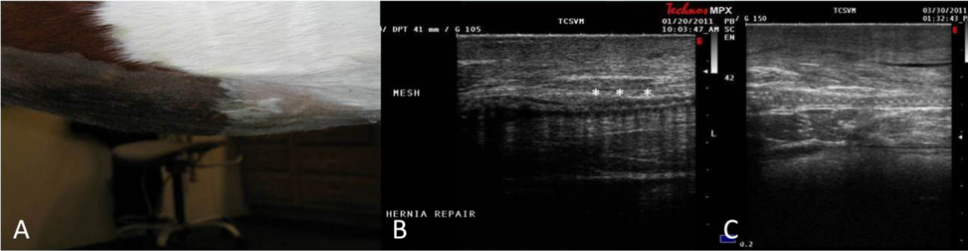
**Postoperative (8-week) image of the ventral midline following mesh hernioplasty (A) with corresponding ultrasound images (B, C) documenting soft tissue incorporation surrounding the silk mesh.** Hypoechogenic shadow artifacts (*) representing the silk mesh are evident. Measurement markings on right side of ultrasound images indicate 0.5-cm intervals.

**Figure 4:**
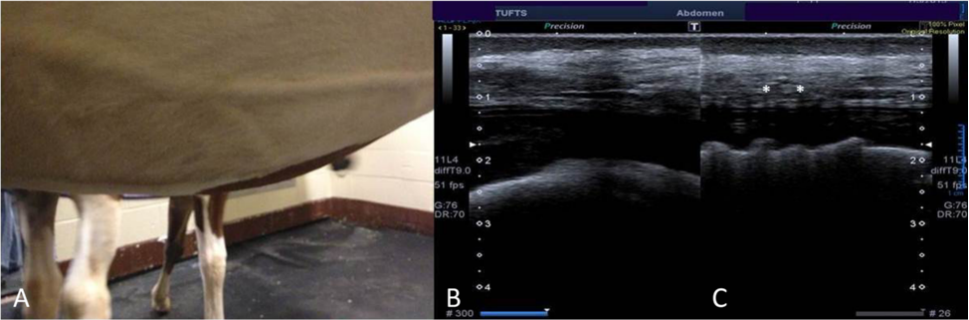
**Postoperative (2 years) image of the ventral midline following mesh hernioplasty (A) with corresponding ultrasound images (B, C) documenting mature fibrous tissue formation throughout the hernioplasty site.** Hypoechogenic shadow artifacts (*) representing remnants of the silk mesh are still evident. Numbers at the edge of the ultrasound images represent 1-cm depth increments.

The mare was discharged from the hospital 3 days postoperatively on a course of parenteral antibiotics (ceftiofur) and anti-inflammatories (flunixin meglumine) with management in an abdominal support bandage. At 8 and 20 weeks postoperatively, ultrasonography was conducted to evaluate healing based on tissue ingrowth of the mesh and fibrous tissue formation around the mesh (Figure 3). No complications were reported at either time point. Furthermore, the ventral abdomen was observed to have a normal contour at both time points. Based on the formation of fibrous tissue surrounding the mesh, the mare was bred 2 years in a row, carrying both foals to full gestation with no complications. Following both foalings, the abdomen has maintained a normal contour with no evidence of hernia recurrence (Figure 4).

## Discussion

Ventral midline hernia formation is a complication following surgical management of abdominal pain in horses with incisional drainage and incisional infection being the largest risk factor for hernia formation [1,2]. In the case presented here, the development of a ventral midline hernia was subsequent to infection of the body wall associated with a previous exploratory celiotomy for treatment of colic. Conventional repair of large ventral midline hernia defects in horses involves subperitoneal implantation of non-absorbable knitted polypropylene or polyester mesh and hernia ring apposition. However, this repair technique has high complication rates (20%–60%), with mortality rates up to 50% due to persistent infection of the implant, rupture of the internal abdominal oblique muscle, and persistent colic, which are all potentially due to the rigidity of synthetic, non-resorbable mesh implants [5,7]. Further concerns regarding currently used non-absorbable mesh implants include tissue erosion, persistent inflammation, infection, and pain following mesh hernioplasty as well as difficulties with potential surgical revision.

Alternative surgical techniques for repair of ventral midline hernias include attempted primary closure of the defect without mesh implantation, subperitoneal mesh placement with fascial overlay, and subcutaneous mesh placement with hernia ring apposition [5-7]. In this case, primary closure of the defect without mesh was previously attempted and likely failed due to the large size of the defect, in addition to the large size of the patient. Subperitoneal placement with fascial overlay involves more aggressive dissection of the hernia sac, allowing more potential exposure of the peritoneal cavity to the mesh implant. Consequences of peritoneal contact with the surface of the mesh include intra-abdominal adhesion of the bowel to the mesh implant, which can lead to chronic colic. In addition, mesh infection would have dire consequences leading to potential septic peritonitis, for which mortality rates range from 40% to 60% in equine patients [9,10]. To help minimize these complications, implantation of subcutaneous mesh following primary closure of the defect has been described [6]. This procedure was not elected in this case, as the significant size of the defect and large patient size would have created a large amount of tension on the ventral midline and body wall, likely causing dehiscence of the primary closure as well as potential failure of the mesh. In addition, as the mesh is placed subcutaneously, there is greater concern for mesh infection.

To our knowledge, this is the first published report describing the use of silk mesh for ventral midline hernioplasty in a large animal. The mesh used in this case is a novel silk-derived bioresorbable scaffold designed to support fibrous tissue ingrowth while allowing long-term bioresorption. Prior to the knitting of the mesh scaffold, the silk is processed to remove the outer sericin coat, which is the major cause of allergenic responses [11]. By removing the sericin, the silk has greater biocompatibility and thus enhanced bioresorption in vivo [8,11]. An earlier version of this silk mesh was evaluated for repair of body wall defects in rodents in comparison to the conventionally used polyester meshes [8]. In contrast to the polyester mesh, the silk mesh had higher reduction in the cross-sectional area over the course of the study, whereas the polyester mesh remained essentially unchanged [8]. Although the silk began to bioresorb, the mesh allowed for significantly greater fibrous tissue ingrowth with biomechanical properties comparable to the polyester mesh at the 3-month termination point of the study [8]. In addition, this study found the inflammatory reaction was similar with both silk mesh and polyester mesh [8]. Subjectively speaking, the silk mesh was extremely easy to work with, less rigid than standard polypropylene or polyester mesh, and less abrasive to the touch. We did not encounter any issues with how the silk mesh handled the suture bites and did not experience any pullout or ripping at the edges during application. Although we believe SERI silk mesh has a soft enough texture not to be abrasive to the bowel if placed intra-peritoneally, we cannot comment on its effect with regards to adhesion formation or bowel irritation.

Gross and colleagues recently published their results of the use of the silk mesh used in this case report for soft tissue support in two-stage breast reconstruction using an ovine model [12]. In their study they were able to demonstrate that the load-bearing responsibility which originally was provided by the mesh was gradually transferred to the new ingrown tissue consisting early on of collagen type 3 and later shifting to collagen type 1. The ingrowth of fibrous tissue resulted in a significant increase in the burst strength of the implant when compared to sham samples [12]. Such increase in strength of the implant has obvious benefits, especially when placed in dependent areas such as in this case report.

In this case report, we aimed to evaluate the potential use of silk mesh in a large horse for ventral midline hernioplasty. In this single case, no complications were encountered when implanting the silk mesh retroperitoneally with hernia ring apposition and closure of subcutaneous tissues and skin. The silk mesh allowed adequate sharing of load between the body wall and mesh, while allowing tissue remodeling and fibrous tissue formation around the defect, as documented by ultrasonography. In our study, remnants of the mesh were visible 2 years following hernioplasty with healthy and organized fibrous scar tissue. In their study, Gross and colleagues were able to document histologically the ingrowth of new collagen fibers, with silk fibrils still visible throughout their 12-month study without evidence of hypersensitivity or immune response [12]. Although we were unable to perform histologic and biomechanical analysis of the repair tissue, the outcome had adequate cosmesis of the ventral abdomen and provided enough biomechanical strength to support the ventral midline of a large horse throughout gestation and parturition of two foals.

## Conclusions

In conclusion, we report the successful use of a novel bioresorbable silk mesh for repair of a large ventral midline hernia in a 600-kg mare. Although this is a single case report, the use of a bioresorbable mesh in a large animal has clinical significance not only for horses but also for humans, as the mesh was able to provide adequate biomechanical strength while allowing for fibrous tissue ingrowth with no complications.
